# Prevalence and correlates of disability among older Ugandans: evidence from the Uganda National Household Survey

**DOI:** 10.3402/gha.v7.25686

**Published:** 2014-11-18

**Authors:** Stephen O. Wandera, James Ntozi, Betty Kwagala

**Affiliations:** Department of Population Studies, School of Statistics and Planning, College of Business and Management Sciences, Makerere University, Kampala, Uganda

**Keywords:** disability, socio-economic vulnerability, older people, non-communicable diseases, Uganda

## Abstract

**Background:**

Nationally representative evidence on the burden and determinants of disability among older people in sub-Saharan Africa in general, and Uganda in particular, is limited.

**Objective:**

The aim of this study was to estimate the prevalence and investigate the correlates of disability among older people in Uganda.

**Design:**

We conducted secondary analysis of data from a sample of 2,382 older persons from the Uganda National Household Survey. Disability was operationalized as either: 1) having a lot of difficulty on any one question; 2) being unable to perform on any one question; or, 3) having some difficulty with two of the six domains. We used frequency distributions for description, chi-square tests for initial associations, and multivariable logistic regressions to assess the associations.

**Results:**

A third of the older population was disabled. Among all older persons, disability was associated with advancement in age (OR=4.91, 95% CI: 3.38–7.13), rural residence (0.56, 0.37–0.85), living alone (1.56, 1.07–2.27), separated or divorced (1.96, 1.31–2.94) or widowed (1.86, 1.32–2.61) marital status, households’ dependence on remittances (1.48, 1.10–1.98), ill health (2.48, 1.95–3.15), and non-communicable diseases (NCDs) (1.81, 0.80–2.33). Gender was not associated with disability among older persons.

**Conclusions:**

Disability was associated with advancement in age, rural residence, living alone, divorced/separated/widowed marital status, dependence on remittances, ill health, and NCDs. Interventions to improve health and functioning of older people need to focus on addressing social inequalities and on the early preventive interventions and management of NCDs in old age in Uganda.

Population ageing is a global phenomenon affecting both developed and developing countries with several implications. First, there is a rise in the prevalence of non-communicable diseases (NCDs) (1–3). Second, a double burden of diseases among older people in Africa, from infectious diseases of poverty and NCDs (4). Third, disability among older persons, which is becoming an important area for research in developing countries (5, 6).

Disability refers to the negative aspects of the interaction between an individual's health condition and his or her contextual factors and environmental factors. It implies having difficulties with activities of daily living (ADL) and instrumental activities of daily living (IADL), and mobility limitations (1, 7). The Washington Group defined disability as having at least a severe difficulty or being unable to perform on any key ADL: sight, hearing, walking or climbing, and remembering or concentrating (8, 9). Broadly, disability negatively affects health, wellbeing, physical functioning, and leads to social exclusion and limited access to healthcare among older people (5, 10, 11).

Globally, disability affects over 1 billion people (12). A multi-country study covering 54 countries using World Health survey data estimated disability at 15% and higher in developing than developed countries (13). In Uganda, the population of older persons increased from 1.1 million in 2002 to 1.3 million in 2010 (14) and is expected to increase from 1.6 million in 2014 to 5.5 million by 2050 (15). According to the 2011 Uganda Demographic and Health Survey (UDHS), the national prevalence of disability in Uganda was 19% (16).

Disability among older people is associated with demographic, socio-economic, and health factors (3, 10, 17, 18). Female *gender* was associated with disability, where older women were at a higher risk of disability than their male counterparts (3, 10, 17, 18). Female gender was associated with disability in Malawi (19), South Africa (20), China (21), and other developing countries (1, 22–24), as well as in the USA (11). Hosseinpoor et al. (24) attributed the gender differentials in disability to socio-economic inequalities between men and women. However, the 90+ Study in Southern California found no relationship between gender and disabilities (25). Advancement in age increases the risk of disability among older people (10, 25) in Nigeria (17), Malaysia (22), Singapore (23), South Africa (26), India (27), Tanzania (18), and in the USA (11).

Rural residence has been found to reduce the risk of disabilities among older persons (28) except in China (21). The prevalence of disability has been reported to be higher among older persons who were not in union (single, divorced, or separated) compared to those who were married (29) in China (21), South Africa (26), India (27), and Uganda (30).

Living alone is associated with health and disability status. In China, older persons who lived alone had poor health status including disability. Those who lived with others are supported emotionally and materially according to the social support theory (29). Poverty status predisposes older persons to the risk of disability (3), especially older women (28) for both developed and developing countries. Poor socio-economic status was a risk factor for disability in the Netherlands (31), Brazil (1), Tanzania (32), and Ghana (33). Lack of/low education level of older persons was associated with disability (1, 10, 28). However, some studies have found no relationship between education and disability (3).

NCDs (e.g., diabetes, stroke, arthritis, and heart disease) are a major underlying cause of disabilities (34). Studies have reported strong associations between NCDs (17, 22, 23, 35), depressive symptoms and dementia (2, 22), stroke (36), diabetes, and disability among older persons (7).

There is limited research on the prevalence and correlates of disability using nationally representative samples among older persons in Africa in general, and Uganda in particular. Available scientific evidence is from World Health Organization (WHO) Study on global AGEing and adult health, Social Assistance Grants for Empowerment (SAGE) (20, 37), or the INDEPTH network data (20). However, many of these studies have not used nationally representative samples. For instance, in Tanzania, a community-based study of the Hai area estimated disability at 6% (18). The few studies that investigated the prevalence and correlates of disabilities among older population in Uganda focused on HIV contexts rather than the general population (30, 37). The aim of this paper was to determine the prevalence and correlates of disabilities in the older population in Uganda, using a nationally representative sample from the 2010 Uganda National Household Survey (UNHS) data.

## Methods

### Data

The study used the 2010 UNHS data. The UNHS used a two-stage stratified sampling. At the first stage, 712 enumeration areas were drawn using probability proportional to size. At the second stage, households were drawn using systematic sampling. A total of 6,800 households were interviewed in the survey (14). Older persons were selected from the sample using the variable age. Persons aged 50 years and older were selected for further analysis, forming a sample of 2,628 older persons. The decision to select persons aged 50 years and above was based on the fact that several studies using WHO and INDEPTH network data define older persons starting at age 50 for African contexts (27, 32, 38, 39).

### Explanatory variables

The UNHS data covered individual and household characteristics – demographic and socio-economic characteristics, disability, health, and housing conditions. Demographic factors included gender (male or female), age group, region, place of residence (rural or urban), living arrangement, relationship to household head, and marital status. Age was recoded into four age categories: 50–59, 60–69, 70–79, and 80+. Region had four categories (1=central, 2=eastern, 3=northern, and 4=western). Living arrangements were recoded into two categories (living alone or with others). Relationship to household head was recoded into three categories (1=head, 2=spouse, and 3=relative). Marital status was recoded into three categories (1=married, 2=separated or divorced or never married, and 3=widowed). Three never married older persons were merged with the separated and divorced category in the data because they were very few.

Socio-economic factors included education level, religion, household poverty status, household major source of earnings, learning technical skill, and ownership of bicycle. Education level was recoded into no education, primary and secondary, or higher education. Religion was recoded into Catholic, Anglican, Muslim, Pentecostal, Seventh Day Adventists, and others. Household poverty status was generated from household expenditures and recoded 1=poor if a household spent less than $1 a day and 0=not poor, if a household spent greater than $1 a day. Household major source of earnings was recoded into farming, wages, and remittances. Learning a technical skill and household bicycle ownership were binary (0=no, 1=yes).

Health-related information was collected on illnesses in the past 30 days preceding the survey, disability, self-reported NCDs (14). Ill health was a binary variable (0=not sick, 1=sick). Self-reporting of diabetes, heart disease, and high blood pressure were used to estimate the prevalence of NCDs, recoded as binary variable (14).

### Outcome variable

Disability was measured by asking six questions on functional limitations on both ADLs and IADLs. ADLs, which mainly focused on body impairments included difficulties in seeing, hearing, walking, and concentrating or remembering. IADLs, which relate to personal care, were measured using difficulties with washing or bathing, feeding, dressing, and toileting as shown below.

1. Do you have difficulty seeing, even if he/she is wearing glasses?
2. Do you have difficulty hearing, even if he/she is wearing a hearing aid?
3. Do you have difficulty walking or climbing steps?
4. Do you have difficulty remembering or concentrating?
5. Do you have difficulty (with self-care such as) washing all over or dressing, feeding and toileting?
6. Do you have difficulty communicating (e.g. understanding others or others understanding him/her) because of a physical, mental, or emotional health condition?

These six questions were originally coded into five categories (1=No, no difficulty, 2=Yes – some difficulty, 3=Yes – a lot of difficulty, 4=cannot perform at all, and 8=don't know). Among the older persons, there was only one ‘don't know’ response on sight disability.

Disability or being disabled was operationalized as either 1) having a lot of difficulty on any of the six indicators; 2) being unable to perform at all on any of the six indicators; or 3) having some difficulty with at least two of the six indicators. This approach to measuring disability has been used in other studies (8, 9, 13). Using these three aspects is better than using ‘having some difficulty on any indicator’ to measure disability (40).

Statistical analyses were done in STATA version 12. In the first place, descriptive statistics (frequency and percent distributions) were analyzed to describe the sample. Second, statistical tests of associations between socio-economic, demographic, and health factors and disability were performed using chi-square tests. The level of statistical significance was set at 95% confidence interval (*p*=0.05). Finally, a full binary logistic regression model was done to predict the correlates of disability among older population in Uganda. Regression diagnostics included a link test and goodness of fit test.

## Results

### Descriptive characteristics of older persons

Table 1 presents the descriptive characteristics of older persons stratified by gender. Overall, there was a higher percentage of older women than older men in the sample. The majority of the older men and women were aged 50–59 years and were from the eastern region. However, the proportion of older women was slightly higher than that of older men as age increased. Nine out of ten older men and women in the sample resided in rural areas and were not living alone. A higher percentage of men than women were household heads and were married.

**Table 1:** Distribution of older persons by demographic, socio-economics, health factors and disability, stratified by gender in Uganda

	Men				Women				All			
			
Variables	Number (*n*)	(%)	% disabled	*p*	Number (*n*)	(%)	% disabled	*p*	Number (*n*)	(%)	% disabled	*p*
Gender												<0.01
Women									1,246	52.3	37.6	
Men									1,136	47.7	27.6	
Age group				<0.01				<0.01				<0.01
50–59	524	46.1	16.1		542	43.5	24.8		1,066	44.7	20.5	
60–69	313	27.6	26.1		356	28.6	37.7		670	28.1	32.3	
70–79	206	18.1	43.7		228	18.3	54.0		433	18.2	49.1	
80+	94	8.2	61.8		120	9.6	64.1		213	9.0	63.1	
Region				0.23				0.44				0.24
Central	278	24.5	27.2		311	24.9	40.4		589	24.7	34.1	
Eastern	371	32.6	31.2		358	28.7	39.1		728	30.6	35.1	
Northern	216	19.1	22.5		253	20.3	33.5		470	19.7	28.5	
Western	271	23.8	27.2		324	26.0	36.5		595	25.0	32.3	
Place of residence				0.03				0.01				<0.01
Rural	1,032	90.8	28.6		1,131	90.7	39.0		2,162	90.8	34.0	
Urban	104	9.2	18.1		115	9.3	24.5		220	9.2	21.4	
Living alone				<0.01				<0.01				<0.01
No	1,022	90.0	25.1		1,145	91.9	35.6		2,167	91.0	30.7	
Yes	114	10.0	49.9		101	8.1	60.1		215	9.0	54.7	
Relationship to household head				0.21				<0.01				0.01
Head	995	87.6	27.2		669	53.7	43.4		1,664	69.9	33.7	
Spouse	72	6.3	21.9		387	31.0	27.6		458	19.2	26.8	
Relative	69	6.1	38.7		191	15.3	37.8		260	10.9	38.0	
Marital status				<0.01				<0.01				<0.01
Married	919	80.9	22.9		477	38.3	26.8		1,396	58.6	24.3	
Divorced/separated/never married	84	7.4	51.5		158	12.7	37.7		242	10.2	42.5	
Widowed	133	11.7	44.8		611	49.0	46.0		744	31.2	45.8	
Education level				<0.01				0.42				0.21
None	713	62.8	25.0		908	72.8	38.8		1,621	68.0	32.7	
Primary	302	26.6	35.0		287	23.0	34.5		589	24.7	34.8	
Secondary +	121	10.7	24.2		51	4.1	34.0		172	7.2	27.1	
Religion				0.58				0.23				0.68
Catholic	527	46.4	25.9		549	44.1	37.1		1,076	45.2	31.6	
Anglican	398	35.0	29.6		448	36.0	37.6		846	35.5	33.8	
Muslim	110	9.7	28.9		96	7.7	45.9		206	8.7	36.9	
Pentecostal	56	4.9	21.5		108	8.7	38.2		164	6.9	32.5	
SDA and others	45	4.0	34.1		44	3.6	25.1		90	3.8	29.6	
Poverty status				0.18				0.18				0.08
Non-poor	883	77.7	26.5		955	76.7	36.5		1,838	77.2	31.7	
Poor	253	22.3	31.5		291	23.3	41.3		544	22.8	36.8	
Major source of earnings				<0.01				<0.01				<0.01
Farming	720	63.4	26.4		731	58.7	37.6		1,451	60.9	32.0	
Wages	330	29.0	23.7		307	24.7	27.0		637	26.7	25.3	
Remittances	86	7.6	53.0		208	16.7	53.5		294	12.3	53.3	
Learnt a trade or technical skill				0.50				0.01				0.02
No	875	77.0	27.1		983	78.9	35.6		1,858	78.0	31.6	
Yes	261	23.0	29.3		263	21.1	45.1		524	22.0	37.2	
Household owns bicycle				0.19				<0.01				<0.01
No	590	51.9	29.5		846	67.9	42.1		1,436	60.3	36.9	
Yes	546	48.1	25.5		400	32.1	28.2		946	39.7	26.6	
Was ill or injured during past 30 days				<0.01				<0.01				<0.01
No	495	43.6	14.9		409	32.8	22.9		904	38.0	18.5	
Yes	641	56.4	37.4		837	67.2	44.8		1,478	62.0	41.6	
Reported an NCD				<0.01				<0.01				<0.01
No	949	83.6	24.5		878	70.4	33.2		1,827	76.7	28.7	
Yes	187	16.4	43.5		368	29.6	48.2		555	23.3	46.6	
Total	1,136	100.0	27.6		1,246	100.0	37.6		2,382	100.0	32.8	

Older men were better off in terms of education levels than older women. The majority of the older men and women were Catholics and were from non-poor households – spent >$1 a day. A higher proportion of older men than women depended on farming and wages. However, a higher proportion of older women than men depended on remittances. An approximately similar proportion of one in five older men and women had learnt a technical skill or trade. More older men's households than women owned bicycles.

Overall, more than half (62%) were sick, with a higher proportion of women than men reporting having an illness in the last 30 days. About two in ten sampled reported at least one NCD (diabetes, heart disease, and high blood pressure) and a third had a disability. Similarly, a higher proportion of women than men reported having an NCD or a disability.

**Fig. 1:**
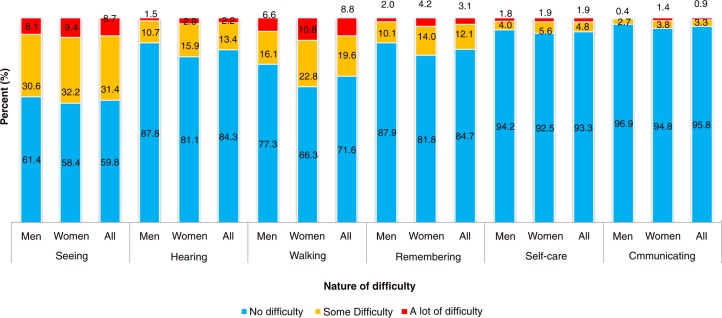
Nature and severity of disability among older people in Uganda.

Figure 1 presents a detailed description of the type and severity of disability among older persons stratified by gender. Sight problem was the leading form of disability followed by walking or climbing difficulties, hearing, and concentrating or remembering difficulties (15%). Self-care and communication challenges were less common among the older population. Another striking feature from Fig. 1 is that older women reported higher prevalence of either some or a lot of difficulty on all of the six domains represented.

### Association between disability and demographic and socio-economic factors

Table 1 also presents chi-square test results for assessment of associations between disability and socio-economic, demographic and health factors. Of all the variables, region, religion, and poverty status were not significantly associated with disability.

Among all older persons, the prevalence of disability was highest among women, oldest old – age 80+, rural residents, those living alone, and those widowed. In addition, disability was highest among those who depended on remittances, learnt technical skill, did not own a bicycle, and reported illness and an NCD.

The same pattern for the prevalence of disability was maintained among older men and women alone, except that the latter showed higher prevalence than the former on various demographic and socio-economic variables (Table 1). The prevalence of disability was higher among older women than men for the oldest old – 80+, rural residents, those living alone and the widowed. In addition, disability was higher among older women than men for those who reported having an illness in last 30 days and an NCD.

### Multivariate results

Table 2 presents the results of multivariable logistic regression of factors associated with disability among older persons stratified by gender. The risk of disability consistently increased with advancement in age for all older persons. Those aged 60–69; 70–79 and 80+ had increased odds of disability compared to those aged 50–59 years. Urban older persons were less likely to be disabled compared to rural older people. This pattern was the same for older women but not older men.

**Table 2:** Results of multivariable logistic regression of disability on socio-economic, demographic, and health-related factors among older people in Uganda

	Men		Women		All	
			
	ORs	95% CI	ORs	95% CI	ORs	95% CI
Age group (rc=50–59)						
60–69	1.64*	[1.09–2.45]	1.46*	[1.06–2.01]	1.51**	[1.18–1.95]
70–79	2.61***	[1.79–3.81]	2.41***	[1.65–3.52]	2.61***	[1.99–3.43]
80+	6.33***	[3.64–11.0]	3.86***	[2.33–6.39]	4.91***	[3.38–7.13]
Urban residence (rc=rural residence)	0.56	[0.30–1.05]	0.56*	[0.34–0.93]	0.56**	[0.37–0.85]
Living alone (rc=living with others)	1.45	[0.76–2.78]	1.58	[0.96–2.61]	1.56*	[1.07–2.27]
Relationship to household head (rc=head)						
Spouse	1.28	[0.47–3.48]	1.42	[0.83–2.43]	1.39	[0.94–2.04]
Relative	1.14	[0.59–2.21]	1.00	[0.67–1.50]	1.00	[0.71–1.40]
Marital status (rc=married)						
Divorced/separated/never married	2.63**	[1.45–4.79]	1.58	[0.86–2.93]	1.96**	[1.31–2.94]
Widowed	1.74	[0.95–3.18]	1.89*	[1.12–3.19]	1.86***	[1.32–2.61]
Education level (rc=no education)						
Primary	1.61**	[1.14–2.28]	0.94	[0.69–1.29]	1.20	[0.96–1.51]
Secondary or higher	1.03	[0.64–1.65]	1.13	[0.56–2.27]	0.98	[0.66–1.46]
Household major source of earnings (rc=farming)						
Wages	1.07	[0.71–1.63]	0.66*	[0.46–0.94]	0.82	[0.62–1.09]
Remittances	2.06**	[1.22–3.48]	1.27	[0.89–1.81]	1.48**	[1.10–1.98]
Learnt a trade or technical skill (rc=no)						
Yes	1.09	[0.76–1.57]	1.43*	[1.03–1.99]	1.24	[0.98–1.56]
Household owns bicycle (rc=no)						
Yes	1.01	[0.72–1.41]	0.63**	[0.46–0.88]	0.79	[0.61–1.02]
Was ill or injured during past 30 days (rc=no)						
Yes	3.00***	[2.13–4.24]	2.19***	[1.61–2.97]	2.48***	[1.95–3.15]
Reported an NCD – diabetes, heart disease or hypertension (rc=no)						
Yes	2.31***	[1.46–3.65]	1.58**	[1.17–2.13]	1.81***	[1.41–2.33]
Gender (rc=women)						
Men					1.09	[0.80–1.48]
Observations	1,241		1,387		2,628	

ORs=odds ratios; CI=95% confidence intervals in brackets

* *p*<0.05

** *p*<0.01

*** *p*<0.001

rc=reference category.

Similarly, all older persons who lived alone compared to those who lived with other people, were more likely to report disability. Living arrangement did not have a significant association among older men and women alone.

A significant relationship existed between marital status and disability for all older persons and had different directions for older men and women. Older people, who were divorced/separated/never married and widowed, were more likely to be disabled compared to those who were married. Among older men alone, having no partner (divorced/separated/never married) increased the odds of disability. Among older women alone, being widowed increased the odds of disability.

Education level was a significant correlate of disability among older men only. Older men with primary education had increased odds of disability.

Older men who depended on remittances had increased odds of disability compared to those who depended on farming. However, older women who depended on wages had decreased odds of disability compared to those who depended on farming. Older women who had a technical skill had increased odds and those whose households owned a bicycle had decreased odds of disability.

Finally, illness during last 30 days and self-reported NCDs were significantly associated with disability among men, women and all older persons. All older persons who were sick and reported an NCD had increased odds of disability. This pattern was observed for both older men and women.

## Discussion

### Burden of disability

The prevalence of disability among older persons in Uganda (33%) is higher than that of the general population (4%) according to the 2002 census (41) and the 2010 UNHS estimate of 16% (14). It is still higher than the 2011 UDHS rate of 20% (16). Studies elsewhere have reported that disability is higher among older population than the younger or general population (13). For example, in Malaysia, disability was reported to be highest (25%) among those aged 60 and older compared to those younger than age 60 (22). A multi-country study using World Health Survey data among 57 countries reported disability ranging from 24% among older men to 40% in older women, which were higher than those of lower ages (24).

### Correlates of disability

As expected, advancement in age was associated with disability among men, women and all older persons. Irrespective of gender, advancement in age leads to the depreciation in the physical functioning of body organs and systems. In addition, advanced age is associated with NCDs that elevate the risk of disability among older people. Our result is in consonance with other studies in Nigeria (17), Ghana (33), rural South Africa (26), Tanzania (32), the United States (25), Malaysia (22), and Brazil (1).

Rural residence was associated with a higher risk of disability than urban residence for all older persons and older women only. This finding is because of rural–urban differentials in socio-economic status and access to healthcare, to the disadvantage of the former. In addition, older persons tend to migrate to rural areas when they either retire or develop some disability and/or a debilitating chronic health condition (42). However, a study in Brazil reported contrasting results that rural older persons were less likely to report disability (1, 28). In addition, there is no clear explanation that can be given for the inconsistent results for older men.

Living alone was associated with disability among all older persons. Living alone, an indicator of vulnerability, has been associated with disability among older people in Uganda (43). According to the social model of disability and intergenerational solidarity (9), older persons living alone are deprived of emotional and physical support from their adult children and the ‘direct health promotional effect of marriage, social support from spouse’ (29). Furthermore, access to adequate healthcare during ill health becomes a challenge for such older persons (9). Subsequently, health conditions that would be prevented such as cataracts might go untreated and lead to total blindness. However, living alone had no significant association with older men and women alone.

Divorced/separated/widowed marital status was associated with disability among men and women. For older women only, being widowed was a bigger problem because about half (49%) of them were widowed. Having no spouse leads to loneliness and depression among older people. Loneliness and depression has been reported to increase the risk of disability among older people (2, 25, 30, 44). Depression resulting from the presence of NCDs, in the absence of emotional support from a partner, can lead to disability also.

Dependence on remittances was associated with disability among older men and all older persons. Being dependent on remittances implies higher likelihood of disability compared to dependence on farming or wages (older women). Those who depend on remittances are disabled and unable to fend for themselves, requiring their relatives to send them assistance to survive. In contrast, older people who are engaged in farming or who receive wages, are still physically fit and hence do not need remittances compared to those who are disabled. Thus, disability calls for significant dependence; the need for care and support from adult children or relatives (45).

Furthermore, disability was significantly associated with ill health and NCDs. Older persons find difficulties in accessing healthcare and are therefore, more likely to be sick. Similarly, reporting at least an NCD (such as diabetes, heart disease, and hypertension), was strongly associated with disability among older persons. Diabetes (2, 7, 35, 46), heart disease (10, 25, 34), and hypertension (2, 10, 30) have been strongly associated with disability among older people.

Although gender was significantly associated with disability at bivariate analysis (Table 1), it became insignificant at multivariate analysis (Table 2), contrary to findings from several other studies (3, 13, 17, 20, 24, 30). For example, in Malawi, older women were more likely to be disabled than older men (19). It is not clear why there were no gender differentials in the prevalence of disability among older persons in Uganda.

### Limitations of data

Several limitations merit discussion. First, self-reported prevalence of NCDs and disability is most likely to be lower than the actual prevalence of either condition, among older people in Uganda. This paper utilized secondary data where indicators of disability were self-reported. The cross sectional nature of the data is limiting. It is difficult to tell whether disability occurred earlier or after the onset of NCDs among older persons.

Second, it was also difficult to distinguish disabilities from birth and those which were a result of occupational hazards and/or a result of advancement in age, though it was clear that increment in age led to a significant increase in the prevalence of disability among older persons. Despite these limitations, the paper contributes to filling the knowledge gap concerning the prevalence and correlates of disability among older persons in Uganda.

### Conclusions and recommendations

Disability was associated with advancement in age, rural residence, living alone, separated/divorced or widowed marital status, and dependence on remittances, sickness, and self-reported NCDs. Therefore, socio-economic vulnerabilities are associated with disability among older persons in Uganda. Surprisingly, gender was not a significant predictor of disability in Uganda.

In order to limit the risk of disability in old age, there is the need for interventions aimed at improving the health of older persons through early prevention and management of NCDs in Uganda. This is because as age increases, the risk of disability from NCDs also increases.

Second, there is a need to reduce socio-economic inequalities among older persons in Uganda. The SAGE, a pilot initiative entailing social cash transfers by the Government in 14 districts, should be rolled out to the entire country in order to promote social protection and improve wellbeing of older persons in Uganda. Special attention should be paid to those who live alone and depend on remittances , as they are more vulnerable compared to other older persons who live with other people.

Third, there is a need for further older persons’ focused research, ideally longitudinal in design that addresses the broad spectrum of ageing and related pertinent issues such as health, living arrangements and intergenerational support in Uganda. This would contribute to a better estimation of NCDs and disability among the older population, and provide a firmer basis for policy advice. Similarly, further investigations to ascertain the association between mental health and disability among older people in African settings should be given priority. The discourse on gender and disability also requires more research especially in sub-Saharan African countries in general and Uganda in particular.
